# Bull selection and management in extensive rangeland production systems of California: a producer survey

**DOI:** 10.1093/tas/txac138

**Published:** 2022-10-05

**Authors:** Megan R Banwarth, Kasey L DeAtley, Craig A Gifford, Tracy K Schohr, Zachary D McFarlane

**Affiliations:** Animal Science Department, California Polytechnic State University, San Luis Obispo, CA 93401, USA; College of Agriculture, California State University, Chico, CA 95929, USA; Extension Animal Sciences and Natural Resources, New Mexico State University, Las Cruces, NM 88003, USA; University of California Cooperative Extension, Plumas-Sierra County, Quincy, CA 96130, USA; Animal Science Department, California Polytechnic State University, San Luis Obispo, CA 93401, USA

**Keywords:** beef bulls, bull management, bull selection, producer survey

## Abstract

California’s approximately 660,000 head of beef cattle are highly dependent on range bulls used to produce offspring that can perform across the state’s diverse ecological regions. Bulls need to be functional on rugged coastal landscapes, rolling foothills, deserts, and in high-elevation terrain. Few data exist that indicate factors related to selection, maintenance, and longevity of bulls used in rangeland landscapes. Objectives herein, were to assess factors influencing bull purchasing, management, and culling decisions of California beef producers. Surveys were mailed to the California Cattlemen’s Association membership (*N* = 1,410) with ~ 16% response rate (*N* = 227). Mean age and years of bull selection experience of respondents was 61 ± 1 yr and 27 ± 1 yr, respectively. Respondents managed cattle on a total of 694,949 hectare of owned, private leased, and leased public rangelands in California and surrounding states. Cow–calf herd size was 333 ± 92 head and bull battery averaged 18 ± 2 head with average bull longevity of 5 ± 1.3 yr. The average price paid for bulls in the last 2 yr was $5007 ± 163.33, while the highest price paid in the last 5 yr was $7291 ± 335.40. Survey responses were used to define current factors driving management after purchase and for subsequent breeding seasons. After bull purchase, 48% of producers turned bulls out directly with females, while 52% held bulls until the following breeding season. Additionally, most producers (70%) did not manage bulls to reduce condition after purchase. Semen quality analysis, a major component of a breeding soundness exam, was evaluated annually by 45% of respondents, while 20% of respondents never evaluated semen quality. Respondents indicated bull age (35%) and structural soundness (29%) as the most common factors for culling bulls. This research shows that despite the variability in operation demographics, there were similarities in beef bull selection and management across the state. Additionally, these data suggest the need for additional research focused on bull selection and management to maximize producer investment in reproduction.

## INTRODUCTION

Beef cattle production systems in California typically depend on grazed rangeland ecosystems for production purposes. The U.S. accounts for 336-million hectares of grazing lands with 48% consisting of rangelands, and California alone encompasses 23-million hectares of rangelands (Schuman et al., 2002; CA Dep. of Forestry and Fire Protection, 2010). Due to the diverse climate, management constraints, and beef cattle operation types across California, producers must utilize unique management strategies to sustainably produce beef on California rangelands. A previous survey of the California Cattlemen’s Association (CCA) membership list indicated that the diversity of ranch structure, management styles, and decision making in California must result in management flexibility of producers, government agencies, and industry partners in order to achieve sustainability goals (Roche et al., 2015). Respondents rated key management practices such as livestock water development, cross fencing, supplemental feeding, and matching genetics and management to the environment as priorities (Roche et al., 2015). Sustainable rangeland management practices are a focus of California producers; however, cattle management and specifically, bull selection and management decision-making are yet to be investigated. Bull selection is an important aspect of any beef operation due to the influx of genetic diversity and improvement predominately in response to the performance of the bull battery (Dhuyvetter et al., 1996). Multiple economic analyses have been performed evaluating the effects of bull performance, phenotypic traits, visually observable characteristics, expected progeny differences (EPD), and other marketing factors on bull prices (Dhuyvetter et al., 1996; Chvosta et al., 2001; Atkinson et al., 2010; Bacon et al., 2017; Boyer et al., 2019). However, most of these analyses have been conducted in the Midwest and do not evaluate how producers manage bulls following purchase. Thus, a survey was conducted with the objectives of assessing factors associated with purchasing, managing, and culling decisions of bulls managed on California rangelands.

## MATERIALS AND METHODS

All procedures used for this survey were approved by the Institutional Review Board (IRB) of California Polytechnic State University, San Luis Obispo (IRB approval number 2019-197). A total of 1,410 surveys were mailed to the California Cattlemen’s Association membership in the form of a paper catalog. Surveys were mailed twice, on 30 January and 18 May 2020. A reminder postcard was sent on 31 May 2020, which provided a Quick Response code and web-based survey link (ucanr.edu/bullsurvey) via the University of California, Davis Qualtrics Online Survey platform. The online version of the survey was available to participants for a 4-mo period from May 2020 to September 2020. A total of 227 individuals responded, consisting of 197 mailed responses from the paper catalog, and 30 responses completed from the online version, resulting in a 16% response rate. However, some surveys were not fully completed, resulting in different response rates for each question. Returned surveys with at least 50% of the responses completed were used in the dataset.

### Survey Questions

A total of 48 questions were categorized into the following focus areas: operation information and rancher demographics (11 questions); general bull selection priorities (17 questions); bull inventory and management (17 questions); and general comments, training/research requests, and bull breeder data sharing requests (3 questions). Questions related to EPDs were Angus-focused in response to the prevalence of educational materials and the breed having the most annual registrations and largest membership in the United States (American Angus Association, 2020).

### Statistical Analysis

All data were managed and recorded in a Microsoft Excel spreadsheet (Microsoft, Redmond, WA). Summary statistics were analyzed in SAS 9.4 (SAS Inst. Inc., Cary, NC, USA) wherein the number of responses, minimum and maximum values, means, medians, standard deviations, and standard errors were calculated for survey responses. Additionally, herd size was used as a demographic to stratify the data using the same categorization as the 2017 Beef Cow–Calf Health and Management Practices in the U.S. survey (USDA, 2020). The following stratification was used to categorize herd-size: small herds (1 to 49 head), medium herds (50 to 199 head), and large herds (200+ head). Pearson correlations and regressions were used to assess relationships among categorical variables to further investigate producer purchasing and management behavior using PROC CORR and PROC REG, respectively. No statistically significant nor strong relationships were detected. Thus, one-way frequency tables and Rao-Scott chi-square goodness-of-fit tests were utilized to examine the frequency counts of categorical responses using PROC SURVEYFREQ.

## RESULTS AND DISCUSSION

### Producer Demographics

**Figure 1.**
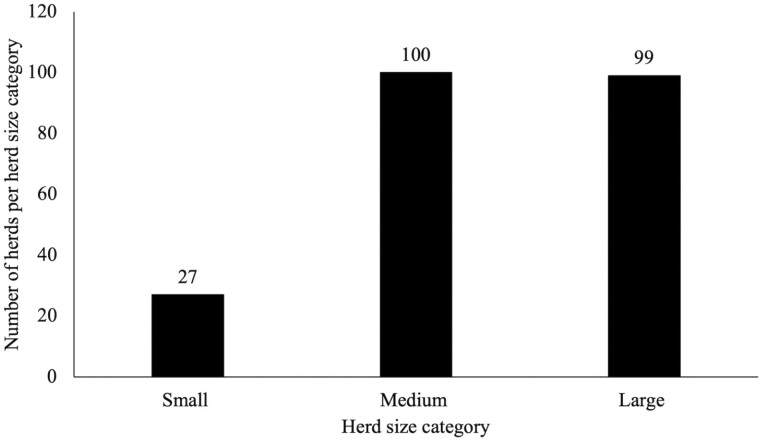
Herd size indicated by respondents (*N* = 226) stratified as small-sized herds (1 to 49 head), medium-sized herds (50 to 199 head), and large-sized herds (200+ head).

Producer respondents (*N* = 226) ranged in age from 20 to 94 yr of age with a mean age of 61 ± 1 yr (Table 1). The average years of experience was 27 ± 1 yr. Current demographic data, particularly the mean producer age of 62 yr, were similar to a previous survey of the same population and survey distribution methodology (Roche et al., 2015). The average herd size was highly variable across the state (Figure 1). Respondents reported an average herd size of 333 ± 42 head, which ranged from 0 head to 7,000 animals (SD = 624). Additionally, the number of stocker cattle exhibited variation with a mean response of 329 ± 107 head with responses ranging from 0 head to 7,000 animals (SD = 1500). The average number of heifers reported by respondents was 55 ± 6 animals. Bull numbers average 18 ± 2 animals, with a range of 0 to 230 animals (SD = 26). The wide range of animals that were reported within herd sizes could be attributed to the fact that some respondents represented smaller herds, while others were some of the largest producers in the state with operations located in multiple states.

**Table 1.** Information about producer and operation demographics

Question topic	Mean	No. of responses	Minimum	Maximum	SD	SEM
Producer age, years	61	226	20	94	15	1
Bull selection experience, years	27	227	1	67	15	1
No. of cattle, total head						
Cow–calf	333	225	0	7,000	624	42
Stocker	329	193	0	13,000	1,500	108
Heifers	55	202	0	520	86	6
Bulls	18	211	0	230	26	2

**Figure 2.**
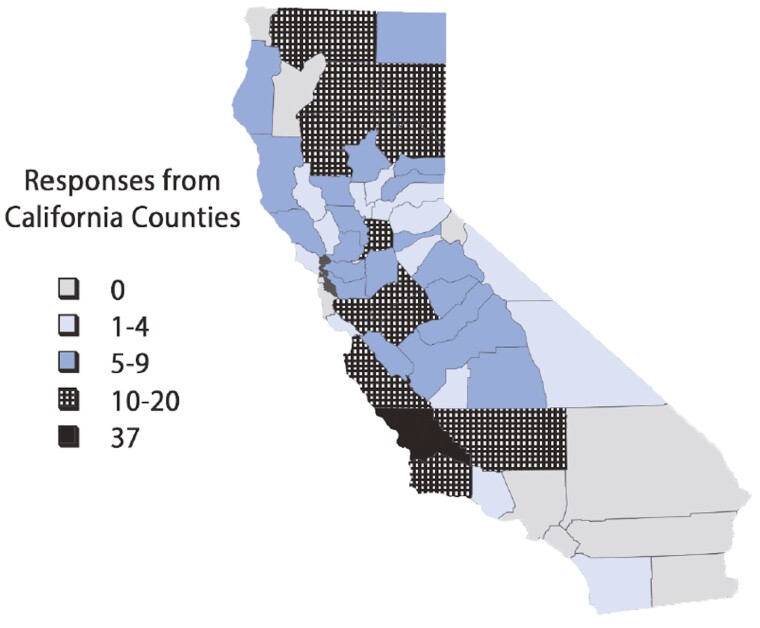
Producer response rates by California counties.

Respondents were predominately male (Table 2; 74%; *P* < 0.0001) with a wide range of education level. Forty-one percent of respondents indicated that a bachelor’s degree was their highest level of education, with 14% of respondents having an advanced degree (e.g., DVM, MA, JD, MD). Other respondents had some college education with no degree (22%) and other various levels of education. Respondents were predominately from California, but some also had operations in Oregon and Nevada (Figure 2). Most producers (63%; *P* < 0.0001) classified their operation as commercial cow–calf, while 20% of producers indicated their operation was a mixture of commercial cow–calf and stocker. Seven percent of respondents classified their operation as purebred/seedstock.

**Table 2.** Frequency of producer responses related to producer background and basic management decisions

Question topic	Frequency, %	No. of responses	SE of %
Sex, % of respondents*		226	
Male	74	168	2.9
Female	26	58	2.9
Education level, % of respondents*		227	
No high school diploma	1	2	0.6
High school diploma	8	18	1.8
College education, no degree	22	49	2.7
Associate’s degree	10	23	2
Bachelor’s degree	41	94	3.3
Postcollege, no degree	4	10	1.4
Advanced degree	14	31	2.3
Operation Type, %*		227	
Commercial cow–calf	63	143	3.2
Stocker	0.4	1	0.4
Seedstock	7	17	1.8
Commercial cow–calf/Stocker	20	45	2.7
Commercial cow–calf/Seedstock	5	12	1.5
Combination (Cow–calf, stocker, seedstock)	4	9	1.3
Acquisition of replacement heifers, %*		223	
Retain	76	169	2.9
Combination	21	46	2.7
Purchase	3	8	1.2
Crossbreeding systems, %*		204	
Rotational breeding	35	72	3.4
Terminal breeding	8	17	1.9
Composite breeding	8	17	1.9
Not applicable, seedstock producer	5	11	1.6
Combination, Rotational/Terminal	2	5	1.1
Not appliable, commercial Black Angus producer	26	54	3
Other Combinations	11	18	1.2
Other	5	10	1.5

*P < 0.0001

### Basic Management Practices of Respondents

Heifer development is an important management consideration that has long-term impacts on profitability for producers because of the associated opportunity and management costs (Funston and Deutscher, 2004; Mulliniks et al., 2013; McFarlane et al., 2018). Respondents (*N* = 223) were asked whether they retained replacement heifers from their own herd or purchased them. Retaining replacement heifers was the predominate choice (76%; *P* < 0.0001), while 21% of respondents indicated a combination of purchasing and retaining replacement females. California producers utilized a wide variety of breeding systems, such as crossbreeding systems. Some version of a rotational breeding system (i.e. two-breed, three-breed, rotation in time, etc.) was the most frequent response (35%; *P* < 0.0001; *N* = 204). Twenty-six percent of producers indicated a commercial Black Angus herd base. Terminal breeding systems (8%) and composite breeding systems (8%) were also represented, while the remainder of respondents indicated a combination (i.e., Rotational/Terminal) of crossbreeding systems (Table 2).

### Factors Influencing Bull Selection and Purchase

Respondents attended an average of 2 ± 0.1 in-person sales per year, with a range of 0 to 10 in-person sales per year (Table 3). Respondents participated in approximately 1 online sale per year on average with a range of 0 to 20 sales per year. Producers (*N*= 201) indicated an average price of US$5,007.00 and a range from US$0 to US$15,000 (SD = US$2,321.30) paid per bull for the last 2 yr (2018 and 2019). However, small-sized herds of 1 to 49 head indicated an average price of US$3,774.00 (SD = US$1,830.70; Table 4). In comparison, medium-sized herds of 50 to 199 head and large sized herds of 200+ head indicated a higher average price of US$5,149.00 (SD = US$2,742.50) and US$5,152 (SD = US$1,842.5), respectively. Respondents (*N* = 208) were also asked the highest price they paid for bulls in the past 5 yr (2014 to 2019); answers ranged from US$800 to US$47,000 (mean = US$7,291.00, SD = US$4,813.80). Small-sized herds indicated an average of US$4,790.00 (SD = US$2,933.90) for their highest-priced bull purchased in the past 5 yr (Table 4). While medium-sized herds indicated an average highest price of US$6,849.00 (SD = US$5,680.20). Large-sized herds indicated an even higher average of US$8,152.00 (SD = US$3,874.30). Similarly, bull numbers increased based on the herd size. Small herds indicated an average of 3 bulls in the battery, medium herds indicating an average of 12 bulls, and large herds indicating an average of 28 bulls. This increase based on herd size in the average price and highest price paid for a bull indicates that herds with more head of cattle were willing to spend more on bull purchases. This increase was in spite of the greater number of bulls purchased for larger herds.

**Table 3.** Information about producer sale attendance and bull purchase price

Question topic	Mean	Median	No. of responses	Minimum	Maximum	SD	SEM
In-person sale attendance, sales/year	2	2	210	0	10	1.4	0.1
Online sale attendance, sales/year	0.5	0	201	0	20	1.9	0.1
Average bull price paid in last 2 yr, $USD	5,007	4,500	203	0	15,000	2321.3	163.33
Highest bull price paid in last 5 yr, $USD	7,291	6,250	208	800	47,000	4813.8	335.4

**Table 4.** Information about bull purchase price stratified by herd size demographic

Question topic	Mean	Median	No. of responses	SD	SEM
Average bull price paid in last 2 yr, $USD			203		
Small herd (1 to 49 head)	3,774	3,200	19	1830.7	419.9
Medium herd (50 to 199 head)	5,149	4,250	91	2742.5	297.5
Large herd (200+ head)	5,152	5,000	93	1842.5	198.7
Highest bull price paid in last 5 yr, $USD			208		
Small herd (1 to 49 head)	4,790	4,250	20	2933.9	13.9
Medium herd (50 to 199 head)	6,849	6,000	94	5680.2	605.5
Large herd (200+ head)	8,152	7,500	94	3874.3	415.4
No. of bulls, total head			211		
Small herd (1 to 49 head)	3	2	22	5.2	1.1
Medium herd (50 to 199 head)	12	6	95	27.7	3.1
Large herd (200+ head)	28	20	94	25.8	3.0
Average bull longevity, years			206		
Small herd (1 to 49 head)	4.1	4	21	1.1	0.2
Medium herd (50 to 199 head)	4.5	4	94	1.5	0.2
Large herd (200+ head)	4.6	4.5	91	1.1	0.1

Bull prices are dictated by the heritability of traits, along with both physical and genetic characteristics of the bulls (Dhuyvetter et al., 1996). A summary of Kansas purebred bull sales in 1993 resulted in a mean value of US$2,306.10 (minimum = US$650, maximum = US$20,000, SD = US$1,272.90). In addition, data from bull sales in Montana, Nebraska, and South Dakota indicate a mean price of approximately US$3,000 (Chvosta et al., 2001). Inflation clearly impacts comparisons among studies referenced and the present study. However, this raises potential opportunities for future economic analyses of bull sale prices in California.

Producers were willing to pay premiums for subjective ratings for muscling, confirmation, and temperament (Dhuyvetter et al., 1996). The increased bull prices in the present study were likely in response to the prices that bull buyers are selling their calves for, as well as in response to other factors related to the individual bull. In Virginia, the sale price of bulls was correlated with the current value of feeder calves (Weaver et al., 2017). These individual bull factors, such as EPDs, sale weight, and frame score, could also be attributed to the increase in bull prices (Brimlow and Doyle, 2014; Boyer et al., 2019).

Most California producers (65%) purchase at least one bull every year (Table 5). However, the indicated average bull longevity only increased 0.5 yr from a small-sized herd to a large-sized herd, thus, suggesting that small-producers do not replace bulls more frequently to reduce the risk of inbreeding (Table 4). Long-yearling (~18 mo of age) bulls were the preference (53%), while 14% of producers preferred yearling bulls, and 15% preferred a combination of yearling and long-yearling bulls. Only 8% of California producers preferred to purchase 2-yr old bulls. In support, bull prices in Kansas had a nonlinear relationship with age, suggesting that producers were willing to pay a premium for older bulls (Dhuyvetter et al., 1996). Older bulls received premium sale prices (Bacon et al., 2017). Studies have indicated buyer preference for long-yearling bulls (McDonald et al., 2010; Brimlow and Doyle, 2014). A survey of U.S. cow–calf producers indicated that approximately 6% of operations use yearling bulls exclusively, while nearly 74% use mature bulls (USDA, 2020). In the western United States, mature bulls were used at a slightly higher rate of 83% (USDA, 2020).

**Table 5.** Frequency of producer responses related to bull selection criteria

Question topic	Frequency, %	No. of responses	SE of %
Annual bull purchase*		213	
Yes	65	138	3.3
No	35	75	3.3
Preferred method of bull purchase*		206	
Sale	47	98	3.5
Private Treaty	31	63	3.2
Combination, private treaty and sale	20	41	2.8
Bred and owned	1	2	0.7
Lease	1	2	0.7
Bull age*		212	
Yearling	14	29	2.4
Long-yearling (~18 mo of age)	53	112	3.4
2-yr old	8	17	1.9
Combination, yearling, and long-yearling	15	32	2.5
Combination, long-yearling and 2-yr old	8	18	1.9
Combination, all three ages	2	4	0.9
Purchase bull for maternal traits*		211	
Yes	83	175	2.6
No	17	36	2.6
Purchase bull for terminal traits*		204	
Yes	69	140	3.3
No	31	64	3.3
Importance of limited bull guarantees*		211	
Yes	73	155	3
No	27	56	3
Out-of-state bull purchases^1^		213	
Yes	51	108	3.4
No	49	105	3.4

*P < 0.0001

^1^= 0.84

Most producers purchased bulls for maternal traits (83%) as well as terminal traits (69%). Limited bull guarantees (e.g., fertility and soundness) were considered important (73% of responses) for bull purchase. The willingness of California producers to purchase bulls from out of state was unclear with 51% indicating affirmation (*P* = 0.84). The importance of bulls as investments for the herd is well-documented; thus, numerous studies have been published outlining producer preferences via hedonic analyses of bull auction data (Bacon et al., 2017). Research has indicated that objectively measured phenotypic traits (e.g., body weight), visually appraised characteristics (e.g., conformation), and bull performance (e.g., average daily gain and/or feed efficiency) are important factors driving the value of bulls (Atkinson et al., 2010; Bacon et al., 2017). Most economic analyses have been conducted in the Midwest, and these data are from sale records. Thus, the present study focused on factors associated with purchasing decisions for California beef producers.

Angus bulls were the predominate breed of preference in California with 67% of producers indicating their predilection for the breed (Table 6). Producers were provided specific selection criteria to list from most to least important for purchasing decisions. The primary selection criteria (Table 7) were structural soundness (63%) and EPD (19%). Additionally, the primary EPD criteria that producers utilized for selection were related to calving ease (birth weight EPD = 36% and calving ease direct = 38%) and weaning weight EPD (17%). The prevalence of producers selecting for EPDs related to calving ease and weaning weight suggests that they prefer bulls that reduce the likelihood of dystocia, yet still have calves that are heavier at the time of weaning. In support, calving ease direct EPD significantly affected Tennessee bull prices every year from the 11 yr of bull sale data analyzed (Boyer et al., 2019). Producers valued birth weight EPD more than the actual birth weight of the bull; however, both actual weight and birth weight EPD significantly affected price (Jones et al., 2008). Many studies have indicated the value of calving ease wherein lower birth weight EPD increased bull price (Jones et al., 2008; Vestal et al., 2013; Brimlow and Doyle, 2014; Bacon et al., 2017). Boyer et al., (2019) were the first researchers to report the positive value of calving ease direct EPD on bull prices.

**Table 6.** Frequency of producer responses related to bull breed preference

Question topic	Frequency, %	No. of responses	SE of %
Breed preference		216	
Angus*	67	144	3.2
Hereford	4	9	1.4
Sim-Angus	5	10	1.4
Simmental	0	0	0
Red Angus	7	15	1.7
Charolais	2	4	0.9
Limousin	0.5	1	0.5
Brangus	1	3	0.8
Combination, Angus and Hereford	4	9	1.4
Combination, multiple breeds	6.5	15	0.7
Other	3	6	1.1

*P < 0.0001

**Table 7.** Frequency of producer responses related to expected progeny difference selection preferences

Question topic	Frequency, %	No. of responses	SE of %
Primary selection criteria*		218	
Structural soundness	63	137	3.3
EPD	19	41	2.7
Genomically-enhanced EPD	2	6	1.1
EPD accuracies	4	8	1.3
Bull’s sire and/or dam	1	2	0.6
Bull breeder reputation/relationship	8	17	1.8
Breeder location	0	0	0
Bull price	3	7	1.2
Primary EPD selection criteria*		209	
Birth weight	36	75	3.3
Calving ease direct	38	79	3.4
Weaning weight	17	36	2.6
Yearling weight	4	9	1.4
Scrotal circumference	3	6	1.2
Milk	2	4	1.0
Primary dollar value index selection criteria*		180	
Beef value ($B)	37	67	3.6
Maternal weaned calf value ($M)	16	29	2.7
Weaned calf value ($W)	29	52	3.4
Cow energy value ($EN)	8	14	2.0
Quality grade ($QG)	5	10	1.7
Yield grade ($YG)	2	3	1.0
Grid value ($G)	3	5	1.2
Use of Angus foot score EPD*		216	
Yes	43	94	3.4
No	31	66	3.1
Unaware	26	56	3.0
Need foot score EPD in other breeds*		204	
Yes	79	161	2.9
No	21	43	2.9

*P < 0.0001

Respondents were also asked about their preferences for dollar value index EPD selection criteria. With Angus being the most popular breed in both California and the United States, the American Angus Association EPD indices were utilized for producers to list from most to least important EPD index for purchasing decisions. Respondents indicated that Beef Value or $B (37%) was the most important EPD value, followed by Weaned Calf Value or $W (29%), and Maternal Weaned Calf Value or $M (16%). Bull carcass characteristics measured via ultrasound were highly valued by Illinois producers and increases in ribeye area, intramuscular fat, and marbling score subsequently increased bull price (Bacon et al., 2017). The present study suggests that beef value is important for bull buyers in California due to respondents indicating that $B is the most important EPD value, which is reflective of carcass and feedlot merit. Producers may be putting more selection emphasis on the beef value EPD in order to pass these performance traits on to weaned calves and/or calves for which they retain ownership postweaning. However, this could also reflect a lack of producer education related to the traits and/or data compiled for calculation of Angus dollar value indices and the potential corresponding changes in their herds. This particular selection pressure for terminal traits may be problematic since most California producers retain their heifers as previously reported. Bacon et al., (2017) also found that weaning weight EPD had a significant influence on bull price wherein bull price increased on average US$9 for every pound over the breed average. Furthermore, recent research has indicated that $W and cow energy value ($EN) had a significant positive effect on bull prices in Idaho (Tejeda et al., 2018). Angus dollar value index EPD selection results presented may have been impacted by the 22.5% of respondents that did not have Angus bulls as their preference. Thus, the responses provided by these producers could have led to a slight skewness of the importance of certain dollar value index EPDs.

California producers manage cattle in rangeland landscapes, often in rugged terrain. Thus, respondents were asked if they utilized the foot score EPD developed by the American Angus Association. The foot score EPD was developed to enable producers to select cattle for structural soundness. Specifically, selection for correct feet and leg structure was assessed with a scoring system of 1 to 9, with 5 representing an ideal structure for foot angle and claw set (Wang et al., 2017). The structural attributes were shown to be moderately heritable traits (Wang et al., 2017). Forty-three percent of producers affirmed the use of the Angus foot score EPD, while 31% of producers did not use the foot score EPD. However, 26% of producers were unaware of the foot score EPD. These data could potentially be skewed by the 29% of respondents that did not indicate Angus as their breed preference. Additionally, the majority of producers (79%) indicated that other breeds should implement a foot score EPD. Data from the present study suggests that further outreach about genetic selection tools for structural soundness in cattle is warranted.

Producers were asked a series of questions to signify their usage of EPD and data for purchasing decisions. The following options were provided: strongly agree, agree, undecided, disagree, and strongly disagree. Producers agreed (83%) that dollar value index EPD values were important for selection (Table 8). Furthermore, the EPD accuracies (79.5%) and the genomically-enhanced EPD values (57%) were also deemed important when selecting bulls to for the herd. EPD explained the variation in bull prices for most of the breeds sold in bull sales in Kansas (Dhuyvetter et al., 1996). EPD from numerous studies evaluating historic bull sale prices showcase the importance of EPD values (Dhuyvetter et al., 1996; Chvosta et al., 2001; Jones et al., 2008; Atkinson et al., 2010; Vestal et al., 2013; Brimlow and Doyle, 2014; Bacon et al., 2017; Boyer et al., 2019). Thus, data from the present study supports previous research indicating the importance of EPD for selection and purchasing decisions. Respondents affirmed the utilization of carcass data for purchasing decisions (79%).

**Table 8.** Frequency of producer responses related to importance of expected progeny differences and performance data for bull selection decisions

Question Topic	Frequency, %	No. of Responses	SE of %
Dollar value index EPD*		217	
Strongly agree	33	72	3.2
Agree	50	109	3.4
Undecided	11	24	2.1
Disagree	5	10	1.4
Strongly disagree	1	2	0.7
Genomically-enhanced EPD*		216	
Strongly agree	18	39	2.6
Agree	39	85	3.3
Undecided	36	78	3.3
Disagree	6	12	1.6
Strongly disagree	1	2	0.7
EPD accuracies*		215	
Strongly agree	26.5	57	3.0
Agree	53	113	3.4
Undecided	16	35	2.5
Disagree	4	9	1.4
Strongly disagree	0.5	1	0.5
Bull carcass data*		215	
Strongly agree	32	69	3.2
Agree	47	100	3.4
Undecided	14	31	2.4
Disagree	6.5	14	1.7
Strongly disagree	0.5	1	0.5
Scrotal circumference*		218	
Strongly agree	26	57	3.0
Agree	54	118	3.4
Undecided	15	32	2.4
Disagree	4.5	10	1.4
Strongly disagree	0.5	1	0.5

*P < 0.0001

Research has shown that buyers consider carcass ultrasound data when making a purchasing decision (Jones et al., 2008; Bacon et al., 2017). As previously mentioned, increased ultrasound measurements of ribeye area and marbling increase sale prices (Bacon et al., 2017). Scrotal circumference (SC) was also indicated as an important factor for bull selection (80%). SC measurements have been utilized for decades. Research has indicated the usefulness of SC to predict semen traits such as semen quality and age at puberty (Geske et al., 1995). Furthermore, moderate heritability was reported in beef bulls (Martinez-Velazquez et al., 2003). Premium sale prices were afforded to older bulls with greater scrotal circumference measurements (Bacon et al., 2017).

California producers were asked questions related to the relevance of individual bull performance data for bull selection and purchase with the following options provided: strongly agree, agree, undecided, disagree, and strongly disagree. Data were unclear regarding the importance of individual bull body weight (BW) on purchasing decisions. Twenty-eight percent of respondents were in agreement with the importance of bull BW, while 27% were undecided and 39% disagreed (Table 9). In contrast, bull sale weight and frame score significantly influenced sale price every year of for 11 yr of Tennessee bull test sale prices (Boyer et al., 2019). Bulls that were larger framed received statistically higher prices than their counterparts, suggesting the importance of weight and size of bulls for purchasing decisions (Bacon et al., 2017). California bull buyers agreed (88%) that bull body condition was an important consideration for bull selection. Bull body condition affects semen quality (Barth et al., 1995) and poor or excessive body condition negatively impacted the probability of breeding soundness examination passage rates (Barth and Waldner, 2002). Data suggest that California producers put emphasis on bull body condition during selection, yet previous research has indicated that excessive body condition affects fertility. In the present study, California producers agreed (80%) that feed efficiency and average daily gain (ADG) were important information for purchasing decisions. In support, bull ADG showed a consistent positive value with bull price data indicating that bull test performance is valued by buyers (Boyer et al., 2019). Most California producers (80%) agreed that bull vaccination program was an important consideration for bull purchase in the present study, which suggests that California producers value herd health programs.

**Table 9.** Frequency of producer responses related to importance of performance data for bull selection decisions

Question topic	Frequency, %	No. of responses	SE of %
Bull bodyweight (BW)*		215	
Strongly agree	6	13	1.6
Agree	22	47	2.8
Undecided	27	59	3.1
Disagree	39	84	3.3
Strongly disagree	6	12	1.6
Bull body condition*		219	
Strongly agree	35	76	3.2
Agree	53	116	3.4
Undecided	10	22	2.0
Disagree	1	3	0.8
Strongly disagree	1	2	0.6
Feed efficiency/average daily gain*		214	
Strongly agree	27	58	3.0
Agree	53	113	3.4
Undecided	16	34	2.5
Disagree	3.5	8	1.3
Strongly disagree	0.5	1	0.5
Bull vaccination program*		217	
Strongly agree	33	71	3.2
Agree	47	103	3.4
Undecided	13	29	2.3
Disagree	6	12	1.5
Strongly disagree	1	2	0.6

*P < 0.0001

Bull marketing has changed in the past decade to include more multimedia marketing. Specifically, bull marketing has evolved to include social media campaigns, videography, and breeder websites. Respondents were asked questions related to the relative importance of bull marketing strategies for their purchasing decisions with the following options provided: strongly agree, agree, undecided, disagree, and strongly disagree. California bull buyers affirmed the importance of sale previews with 82% of respondents in agreement (Table 10). The relative importance of bull pictures and videos were less clear with 45% and 41% in agreement, respectively. Thirty-three percent of producers indicated that they were undecided on the importance of bull pictures and videos to influence selection and purchasing decisions. A study of Kansas bulls sales reported that bulls with pictures included in sale catalogs received approximately 28% higher prices when compared with their nonpictured counterparts (Dhuyvetter et al., 1996). However, sale order also influenced prices resulting in a reduction in the value of bull pictures later during sales (Dhuyvetter et al., 1996). California producers do not utilize social media to influence bull purchasing decisions with 64% of respondents designating that they disagreed with the importance of social media in bull marketing. Farmer age influenced willingness to utilize social media in the United Kingdom wherein social media usage drastically declined in farmers who are 50 yr and older (Morris and Penri, 2017). Thus, respondent age in the present study likely contributed to the lack of importance for bull marketing via social media. In addition, livestock publication/magazine advertisement was also not a strong influence on purchasing and selection decisions. Thirty-one percent of respondents were undecided and 42% indicated they disagreed with the importance of bull marketing in livestock publications. California bull buyers agreed (60%) that they were loyal to specific breeders. In support, research has shown that breeder reputation influenced sale price (Dhuyvetter et al., 1996; Jones et al., 2008). Additionally, research suggests that sale location and other marketing influences that may be difficult to assess determined bull prices (Dhuyvetter et al., 1996). Bulls that sold early in the sale brought higher prices (Bacon et al., 2017). Thus, other factors related to bull marketing that were not assessed in the present study influence bull selection and purchasing decisions.

**Table 10.** Frequency of producer responses related to importance of bull marketing for selection decisions

Question topic	Frequency, %	No. of responses	SE of %
Sale preview*		216	
Strongly agree	60	130	3.3
Agree	32	70	3.2
Undecided	4	8	1.3
Disagree	3	7	1.2
Strongly disagree	1	1	0.5
Bull pictures*		219	
Strongly agree	7	16	1.8
Agree	38	83	3.3
Undecided	33	72	3.2
Disagree	19	41	2.6
Strongly disagree	3	7	1.2
Bull videos*		218	
Strongly agree	13	29	2.3
Agree	28	60	3.0
Undecided	33	73	3.2
Disagree	22	47	2.8
Strongly disagree	4	9	1.4
Social media*		219	
Strongly agree	3	7	1.2
Agree	6	13	1.6
Undecided	27	59	3.0
Disagree	40	88	3.3
Strongly disagree	24	52	2.9
Magazine advertisement*		216	
Strongly agree	3	7	1.2
Agree	24	52	2.9
Undecided	31	67	3.2
Disagree	30	65	3.1
Strongly disagree	12	25	2.2
Breeder loyalty*		215	
Strongly agree	18	38	2.6
Agree	42	90	3.4
Undecided	14	30	2.4
Disagree	25	54	3.0
Strongly disagree	1	3	0.8

*P < 0.0001

### Bull Management after Purchase and for Breeding

Producer management preferences for bulls prior, during, and after the breeding season have not been well-documented. California producers indicated that they utilized 1 breeding season per year with an average length of 3.5 mo (Table 11). In addition, producers indicated 5 yr as the average length of time that bulls were used on their operations.

**Table 11.** Information about bull management during the breeding season and bull longevity

Question Topic	Mean	No. of responses	Minimum	Maximum	SD	SEM
Number of breeding seasons per year	1.4	205	1	4	0.5	0.04
Length of breeding season, months	3.5	165	2	12	1.7	0.1
Average bull longevity, years	5	206	2	10	1.30	0.1

Survey respondents were asked to choose their typical bull turnout timeframe after purchase. Producer preference was unclear (*P* = 0.54, Table 12). Forty-eight percent of bull buyers turn bulls out directly with females (within 30 d), while 52% hold bulls until the following breeding season after purchase. Producers were also asked the frequency at which semen quality was evaluated. Twenty-two percent of respondents never evaluated semen quality during breeding soundness exams after purchasing a bull, while 43% assessed semen quality annually during breeding soundness exams, and 21% evaluated prior to the start of the breeding season. Bulls are rarely sterile, but subfertility can be an issue even if bulls pass a breeding soundness exam; therefore, a comprehensive analysis of sperm function at the whole-animal, molecular, and cellular levels are recommended to predict bull fertility (Kastelic and Thundathil, 2008). The U.S. Department of Agriculture cow–calf management survey reported that 62% of operations performed a semen test in the western region (USDA, 2020). Operations with larger herd sizes (200+ head) conducted semen evaluations at a greater rate when compared with medium (50 to 199 head) and small (1 to 49 head) operations. Approximately 20% of all U.S. cow–calf operations evaluate semen quality (USDA, 2020). Surprisingly, 34% of respondents in the current study indicated that they use reproductive technologies like artificial insemination. Previous reports (USDA, 2020) found nearly 12% of U.S. cow–calf producers utilize artificial insemination, while artificial insemination was practiced by only 8% of producers in the western region (USDA, 2020). Data in the present study showcased a lower percentage of operations evaluating semen quality in California when compared with cow–calf operations in the western United States. Thus, opportunities for producer education with respect to reproductive management are warranted for California producers.

**Table 12.** Frequency of producer responses related to bull management for the breeding season

Question topic	Frequency, %	No. of responses	SE of %
Bull turnout timeframe^1^		211	
Immediately or within 30 d	48	101	3.4
Hold bulls until following breeding season	52	110	3.4
Frequency of semen evaluation*		219	
Never	22	49	2.8
Annually	43	94	3.4
Bi-annually	3	7	1.2
Beginning and end of breeding	0	0	0
Prior to start of breeding	21	46	2.8
Annually at the start of breeding	5	11	1.5
Other	6	12	1.5
Trichomoniasis testing*		213	
Yes	63	135	3.3
No	37	78	3.3
Breeding Season*		212	
Fall	42	90	3.4
Spring	23	48	2.9
Multiple	35	74	3.2
Average bull:cow ratio*		211	
1 bull:25 cows	50	105	3.5
1 bull:30 cows	17	36	2.6
Other	33	70	3.2
Use of artificial insemination*		211	
Yes	34	71	3.3
No	66	140	3.3

*P < 0.0001

^1^= 0.54

Trichomoniasis (trich) testing was considered a priority for California producers with 63% of producers confirming testing (Table 12). Western region cow–calf producers tested for trich at a greater rate (63.7 ± 4.7%) when compared with all U.S. cow–calf producers (53.6 ± 2.8%). Trichomoniasis is a prevalent disease in California herds with 380 total cases reported between 2015 and 2019, which resulted in California having the third highest case rate out of all 50 states between that time periods (Martin et al., 2021). Trichonomiasis negatively affects the productivity of cattle by reducing conception rates, reducing the number of calves produced, longer calving intervals, and higher culling rates of bulls (Rae and Crews, 2006; Michi et al., 2016). Fifty percent of respondents use a bull to cow ratio of 1 bull:25 cows, while 33% indicated “Other” for their response with most producers using a lower ratio.

Questions related to bull maintenance and management were related to a variety of factors such as health, off-season management, and culling decisions. Bull vaccination was a high priority (91%) for respondents in the current study (Table 13). In addition, California producers recognized the value of bull parasite control with 88% of producers in affirmation. Typically, bulls are fed to increase body condition prior to sale (Barth and Waldner, 2002). Thus, producers were asked how bull body condition was managed after purchase. Most respondents (70%) indicated that they did not manage bulls to reduce condition prior to the breeding season. As previously reported, most producers in the present study consider bull body condition important for selection and purchase decisions. However, changes in body condition during the breeding season may have an influence on semen quality and is an important management consideration for producers after purchasing a bull (Barth and Waldner, 2002). Additionally, respondents were asked to identify how bulls were managed in the off-season (i.e., outside of the breeding season). Fifty-one percent of producers had a separate bull pasture for grazing, while 21% of respondents utilized a combination of bull pasture grazing and supplemental hay. Producers recognized the importance of mineral supplementation, as 93% of respondents indicated use of a supplementation program (*P* < 0.0001; *N* = 210). Recent research indicated that regionality influences the mineral status of California beef cattle herds (Davy et al., 2019). Overall, California producers seem to be aware of mineral deficiencies in their specific region of California. Along with respondents being asked the average longevity of bulls in the herd (mean = 5 yr), producers in the current study were also asked to identify their primary reason for culling bulls. Bull age (35%), soundness (29%), and injury (11%) were the primary justification for culling decisions. In 2017, approximately 93% of cows were bull-bred exclusively, and 76.8% of heifers were bred only by bulls in the United States (USDA, 2020).

**Table 13.** Frequency of producer responses related to bull management and culling decisions

Question topic	Frequency, %	No. of responses	SE of %
Bull vaccination*		205	
Yes	91	186	2.0
No	9	19	2.0
Parasitic control*		209	
Yes	88	184	2.3
No	4	8	1.3
Sometimes	8	17	1.9
Bull condition management*		215	
Yes	30	65	3.1
No	70	151	3.1
Bull management in off-season*		218	
High-energy diet	0	0	0
Grazing bull pasture	51	111	3.3
Feed hay/supplemental forages	6	12	1.5
Run with bred cows	5	11	1.5
Combination, bull pasture and hay	21	45	2.7
Combination, bull pasture and bred cows	5	12	1.5
Combination, bull pasture/hay/bred cows	4	9	1.4
Other combinations	8	18	1.9
Mineral Supplementation, %*		210	
Yes	93	196	1.7
No	7	14	1.7
Primary reason to cull bulls*		210	
Age	35	73	3.3
Soundness	29	60	3.1
Injury	11	23	2.2
Fertility	10	21	2.1
Inbreeding	3	8	1.3
Temperament	3	6	1.2
Body condition	3	6	1.2
Other	6	13	1.7

*P < 0.0001

Collectively, the results from the present study indicate that research evaluating bull management before, during, and after the breeding season is warranted. The frequency of semen evaluation data were troubling. Future outreach efforts should include discussions of best practices in bull management for success in the breeding season is necessary. Calving ease EPD values were reported to still be the most important genetic prediction tools for California bull buyers. These data suggest that future trainings in Angus foot scoring and potential for structural soundness genetic evaluations in other breeds are warranted. Future surveys and research should ascertain further details about bull management. Bulls are likely managed differently in different regions of California. Ultimately, this is the first study to investigate bull management strategies and culling decisions. A follow-up survey should incorporate qualitative data with producer interviews to provide further insight into bull management strategies.
